# Death of Prof. Francis G. Smith

**Published:** 1878-06

**Authors:** 


Death of Prof. Francis G. Smith.—Dr. Francis Gurney Smith, Professor of the Institutes of Medicine in the University of Pennsylvania, died in his native city, Philadelphia, on April 13th, at the age of 60. Since his graduation in medicine in 1840, he has held a prominent place in the profession. He was one of the compilers of "Neill & Smith's Compendium of Medicine," was editor of the *Philadelphia Medical Examiner* for nine years, and of the American editions of Carpenter's and Marshall's works on Physiology, and the translator of "Barth and Rogers' Manual of Auscultation and Percussion.".—*Hospital Gazette.*

